# Metabolomics in Plants and Humans: Applications in the Prevention and Diagnosis of Diseases

**DOI:** 10.1155/2013/792527

**Published:** 2013-08-06

**Authors:** Diego F. Gomez-Casati, Maria I. Zanor, María V. Busi

**Affiliations:** ^1^Centro de Estudios Fotosintéticos y Bioquímicos (CEFOBI-CONICET), Universidad Nacional de Rosario, Suipacha 531, 2000 Rosario, Argentina; ^2^IIB-Universidad Nacional de General San Martín (UNSAM), 25 de Mayo y Francia, San Martín, 1650 Buenos Aires, Argentina; ^3^Instituto de Biología Molecular y Celular de Rosario (IBR-CONICET), Universidad Nacional de Rosario, Suipacha 531, 2000 Rosario, Argentina

## Abstract

In the recent years, there has been an increase in the number of metabolomic approaches used, in parallel with proteomic and functional genomic studies. The wide variety of chemical types of metabolites available has also accelerated the use of different techniques in the investigation of the metabolome. At present, metabolomics is applied to investigate several human diseases, to improve their diagnosis and prevention, and to design better therapeutic strategies. In addition, metabolomic studies are also being carried out in areas such as toxicology and pharmacology, crop breeding, and plant biotechnology. In this review, we emphasize the use and application of metabolomics in human diseases and plant research to improve human health.

## 1. Introduction

Metabolomics is the comprehensive, nonbiased, high throughput analysis of complex metabolite mixtures allowing ideally the identification and quantification of every individual metabolite. Metabolomics has emerged as a functional genomics methodology that contributes to our understanding of the complex molecular interactions in biological systems [1]. As such, metabolomics represents the logical progression from large-scale analysis of RNA and proteins at the systems level [2, 3] but unlike transcripts and proteins, the highly diverse molecular identity of metabolites relies on sophisticated instrumentation such as mass spectrometry (MS) and nuclear magnetic resonance spectroscopy (NMR) [4] to be determined.

Metabolites are the end products of cellular regulatory processes, and their levels can be regarded as the ultimate response of biological systems to genetic or environmental changes [5]. Metabolites are also highly dynamic in time and space and show an immense range of structures raising the challenges for analytical procedures in their measurement [6]. 

Metabolomics allows for a global assessment of a cellular state within the context of the immediate environment, taking into account gene expression, genetic regulation, altered kinetic activity and regulation of enzymes, and changes in metabolic reactions [7–9]. Thus, metabolomic studies compared with genomics or proteomics reflect changes in phenotype and function of a particular tissue, or organism. Thus, the *omic* sciences are used in a complementary manner to carry out studies in order to characterize a phenotype. The change in the expression of genes and proteins will surely produce changes in the metabolic profile of a cell, tissue or organism [7, 10]. Moreover, metabolomic determinations offer the same advantages as proteomic and transcriptomic techniques: they all have the ability to assay biofluids and are relatively inexpensive, rapid, and automated techniques once start-up costs are taken into account.

**Figure 1:**
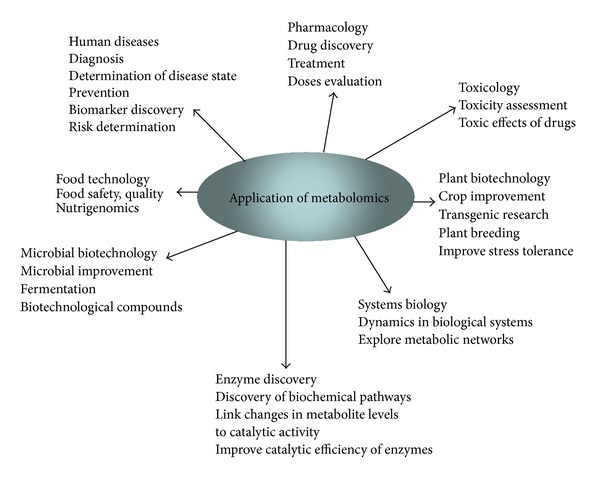
Applications of metabolomics.

Metabolomics was first applied to the study of toxicology and pharmacology, inborn metabolic errors, and nutrition [10]. There is currently great interest in the application of metabolomics to characterize different pathological states of human diseases like cancer, diabetes, autoimmune, and coronary diseases. Moreover, metabolomics can provide valuable tools in a wide range of applications, including microbial biotechnology, food technology, pharmacology, toxicology, enzyme discovery, systems biology, and plant biotechnology (Figure 1). Current research in plants makes extensive use of metabolomics due to the benefit produced on human health of various products of plant origin, including food, pharmaceuticals, and industrial raw materials, as well as its use in plant breeding and nutrition assessment. In addition, the vast chemical diversity of plants compared with animals and microorganisms also contributes to the prominence of metabolomics in plant research [11].

Recent technological progress in NMR spectroscopy and MS, the two most accepted methods used in the measurement of metabolites, has improved the sensitivity and spectral resolution of analytic assays on metabolomic samples in attempts to achieve a comprehensive biochemical measurement. Although not all strategies used in metabolomics are universally accepted it is however possible to summarize the most popular ones [19–22] (see Table 1), including metabolite target analysis, metabolite profiling, metabolomics, metabolite flux analysis, and metabolic fingerprinting (and footprinting). 

## 2. Metabolomics in Human Diseases 

One of the most important applications of metabolomics in the study of human diseases is in the field of oncobiology. Because tumor cells are highly proliferative and have a high transcription and translation rates, as well as a higher energy demand, they have special metabolic requirements when compared to normal cells and frequently lose many regulatory functions [35]. Thus, one of the greatest challenges in medicine is the use of metabolomics in predicting the appearance of tumor cells. Initially, putative metabolic biomarkers for cancer detection and/or assessment of efficacy of anticancer treatment are discovered in preclinical analyses, followed by the validation of these biomarkers in biofluids (blood, urine, prostatic secretions, etc.) [36]. At present, different metabolites have been identified and proposed that would serve as markers for several tumor processes and other diseases (see Table 2). However, in most cases the combination of metabolomics with other genomic and/or proteomic techniques is extremely useful for both prevention and diagnosis of cancer. 

One of the most frequent tumors with an increasing incidence in humans is the hepatocellular carcinoma (HCC). Liu et al. [37] reported the identification of cytokine biomarkers using antibody microarrays. They identified several protein markers such as a macrophage-derived chemokine and macrophage-stimulating protein differentially expressed in patients with liver carcinoma. In addition, different studies were performed in order to obtain a metabolite profile in patients with HCC. Using ultraperformance liquid chromatography-electrospray ionization and TOF mass spectrometry, Patterson et al. [38] have described an alteration of lipid metabolism in these patients, with an increase of glycodeoxycholate, deoxycholate-3-sulfate, and bilirubin. Moreover, the reduction in the levels of lysophosphocholine correlated with the appearance of HCC. Other metabolites found to be altered in liver cancer were polar compounds especially those from the amino acid group such as arginine, proline, alanine, lysine, and aspartate [39]. In addition, an increased level of some amino acids (including alanine) was found in other tumors such as brain tumors, gliomas, and neuroepithelial tumors [40]. Recently, it was described that compounds such as choline, phosphocholine, myoinositol, glycine, taurine and glycerophosphocholine are altered in some types of breast cancers. In this type of diseases, usually other molecules such as the estrogen receptor and the Human Epidermal Growth Factor Receptor 2 (*Her2/neu*) are widely used as biomarkers for prognostic or predictive purposes [10]. On the other hand, myo-inositol levels were also found to be increased in prostate cancer, colon adenocarcinoma, and ovarian carcinoma [41, 42]. 

The use of stable isotopes in cancer research has also been reported. Stable isotopes are nontoxic compounds such as ^13^C-labeled metabolites which can be used to investigate metabolic pathways in normal or highly proliferative cells [43]. Such isotopes can be distinguish easily by NMR or MS and provide information about changes in the regulation of biochemical pathways between cancer and noncancer cells, leading to the development of new diagnostic tools. 

However, there are several gaps in the knowledge of the cancer metabolome. The metabolite profile could vary among the different tumor types making it difficult to generalize findings across tumor groups. There are also technical difficulties encountered while performing metabolomic assays that may hinder the characterization of a tumor metabolome, including sample-to-sample variation, the sensitivity and the physiological status of the tumor [10].

Another application of metabolomics in the field of human health includes nutrigenomics. The term nutrigenomics is associated with the interaction of the diet and the genes and reflects the change in gene expression that takes place after the exposure to different nutrients. The identification of certain compounds that would have the ability to act on the expression of target genes is extremely important in preventing diseases such as cancer. Moreover, this interaction could influence the absorption, digestion, and the elimination of metabolites [44]. Thus, the metabolomic approach may allow the discovery of bioprotective foods [20].

Finally, metabolomics became indispensable for the identification and prevention of coronary heart disease. The recognition of a myocardial ischemic episode is important for both the diagnosis and therapy of the disease [45]. At present, several protein and enzyme biomarkers are used for the identification of patients with risk of coronary diseases such as reactive C-protein, tumor necrosis factor *α* (TNF-*α*), receptors types 1 and 2 (sTNF-R1 and sTNF-R2), and interleukin-6 [46]. However, numerous efforts are underway to identify metabolites that may also serve as markers of disease using metabolomic techniques. Sabatine et al. (2005) reported an alteration in the levels of several metabolites that could serve as biomarkers of myocardial ischemia. Plasma levels of lactic acid and also metabolites involved in skeletal muscle AMP catabolism such as hypoxanthine and inosine as well as alanine were found to be increased after myocardial injury [47]. On the other hand, several metabolites such as Krebs cycle intermediates, uric acid, and GABA yielded significant but discordant changes in controls and cases. Mayr et al. (2008) used a combined metabolomic and proteomic approach to study patients presenting persistent atrial fibrillation. Those results showed a rise in beta-hydroxybutirate levels with an increase in different ketogenic amino acids and glycine [48]. The metabolomic data correlates with the increase in 3-oxoacid transferase, a key enzyme involved in ketolytic energy production. Recently, Shah et al. (2010) characterized the metabolic profile of patients with cardiovascular disease using 69 metabolites [49]. Different acylcarnitines as well as several amino acid levels including leucine, isoleucine, glutamate, glutamine, proline, and methionine were found to be altered in patients when compared to control groups [49]. In addition, it has been reported that the level of the metabolite dicarboxylacylcarnitine could be predictive of cardiovascular disease. More recently, using plasma metabolic fingerprint, it has been possible to classify patients with aortic abdominal aneurysm (AAA) and to predict the disease stage. In this assay, sphingolipids, lysophospholipids, cholesterol, acylcarnitines, and guanidinosuccinic acid were proposed as markers of AAA [50]. 

Although many efforts have been made in order to find new metabolic markers for the prevention of cardiac diseases, the metabolomic approach is still under development. To better understand metabolic pathways alterations, including changes in metabolite levels and gene transcription taking place in patients with risk of cardiac disease, it is necessary to use integrated approaches, combining biomarkers from genomics, proteomics, and metabolomics. This will result in a better picture and therefore a better selection of the potential biomarkers for prevention, diagnosis, and risk prediction of heart disease [51].

## 3. Metabolomics and Its Applications in Plant Biotechnology

Metabolomics originally developed from metabolic profiling. In the early 1970's GC-MS technologies were used to analyze steroids, acids, and neutral and acidic urinary drug metabolites [52–54]. Soon afterwards, the concept of using metabolic profiles to screen, diagnose, and assess health began to spread [55, 56]. However, it was not until the early 1990's that metabolite profiling was used to study plant organisms [14, 57, 58]. 

Plant metabolites are involved in many resistance and stress responses and also contribute to the color, taste, aroma, and scent of fruits and flowers [2]. As we mentioned previously, the biochemical phenotype of an organism is the final result of interactions between the genotype and the environmental stimuli; but it is also modulated by intracellular physiological fluctuations that are part of homeostasis [3]. Thus, the simultaneous identification and quantification of metabolites is necessary to understand the dynamics of the metabolome, analyze fluxes in metabolic pathways and decipher the role of each metabolite following various stimuli [59]. The challenge of metabolomics is to find changes in biochemical pathways, and metabolic networks that might correlate with the physiological and developmental phenotype of a cell, tissue, or organism [2, 3].

One of the greatest achievements of plant biology is the completion of the whole genome sequences of model plants such as *Arabidopsis thaliana* and rice. In Arabidopsis ~27000 genes were predicted based on nucleotide sequence information; however, only half of these genes have been functionally annotated based on sequence similarity to known genes, and among these the function of only ~11% has been confirmed with direct experimental evidence [60]. The elucidation of unknown genes function is therefore currently a major challenge in plant research. Because there is very little information on the number of genes in a particular gene family of a nonmodel plant, it becomes necessary to know the profile of expression of these genes under different conditions and stimuli. The integration of metabolomics with transcriptional profiles can provide clues for the identification of the functions of the unknown genes, regardless of whether they are from model or nonmodel plants [61].

Plants produce more than 200,000 metabolites, many of which play specific roles in allowing adaptation to specific ecological niches [5, 62]. Therefore, the main problems encountered when characterizing the plant metabolome have to do with the fact that in comparison to the proteome or transcriptome, the metabolome is highly complex in nature, due to the enormous chemical diversity of the compounds. In addition, there is a wide range of metabolite concentrations, which can vary over nine orders of magnitude (pM to mM). These large variations in the nature and the concentration of analytes to be studied provide challenges to all the analytical technologies employed in metabolomic strategies [63].

Using metabolomics, it is possible to identify pathways responsible for the production of important food metabolites that could be important in improving human health. There are several examples where the modification of certain metabolic pathways led to the production of plants with an increased nutritional value. This is the case of Golden Rice (GR), which is genetically modified rice that accumulates *β*-carotene in the endosperm [64]. The production of this variety of rice allowed alleviating vitamin A deficiency, a major nutritional problem worldwide. The nutritional value of GR was later improved by the overexpression of a phytoene synthase gene leading to the obtention of the GR2 variety, which accumulates higher amounts of carotenoids (84% of the total is *β*-carotene) [65]. Mehta et al. (2002) were able to express an S-adenosylmethionine decarboxylase gene under the inducible E8 promoter in tomato. The transgenic variety shows higher levels of different polyamines during fruit ripening, including spermidine and spermine, leading to an increase in the metabolite lycopene, which prolonged vine life and enhanced fruit juice and nutrient quality [66].

Other examples include the engineering of plants to enhance anthocyanin content. Anthocyanins are flavonoids, a class of pigments that contribute to the colors and antioxidant qualities of plants. Moreover, these metabolites have been associated with the protection against several human diseases, but their natural levels in plants are inadequate to confer optimal benefits. Recently, it has been reported that the expression of two transcription factors in tomato led to the accumulation of higher quantities of anthocyanins at concentrations comparable to those founded in high antocyanin-containing plants such as blackberries and blueberries [67]. The new variety has an intense purple coloration and also a 3-fold enhanced antioxidant capacity. In addition, this study also reported an extension of the life-span of cancer susceptible mice which were fed with a diet supplemented with this tomato variety.

 Plant metabolomics is being increasingly used for understanding other processes such as the cellular responses to stress conditions. The metabolic readjustment to sulfur deficiency is an example of this [74]. A close relationship was found between sulfur assimilation, nitrogen, lipid, and purine metabolism and enhanced photorespiration. Metabolomics has also been applied to the study of the cold stress response [75]. Other applications include metabolic engineering of biochemical pathways, gene function discovery, and engineering pathways for pharmaceuticals production (Table 3) [76].

## 4. Methods Used for the Determination of Plant Metabolomes

The basic instrumentation useful for the evaluation of plant metabolome includes direct spectroscopy, chromatography (fingerprinting), or high-performance chromatography (GC or HPLC) combined with spectroscopy (IR, NIR, MS). The coupling of chromatographic methods with MS can substantially increase the depth of metabolome coverage, add an additional dimension for metabolite identification and enhance the biological context through the more rigorous identification of a greater number of metabolites [77].

GC-MS is one of the most widely used analytical techniques in plant metabolomics. It is utilized to analyze a qualitatively and quantitatively wide range of volatile and/or derivatized nonvolatile metabolites with high thermal stability. After separation, the eluted metabolites are identified by mass spectrophotometers. This technique has high analytical reproducibility and lower costs compared to other hyphenated techniques, such as LC-MS or LC-NMR [15, 77]. Alternatively, direct injection MS analysis may also be applied for the phenotyping of plants, that is, Fourier transformed-MS (FT-MS) provides ultimate limit of detection and mass measurement precision to enable metabolomic analyses [77, 78].

 In the last years, the use of LC-MS in plant metabolomics has been increasing. LC-MS is more suitable than GC-MS for labile compounds as well as for those that are difficult to derivatize. The application of LC-MS in plant metabolomics includes the description of the tomato metabolome database (MoTo DB) [79], the identification of the accumulation of oxylipins after wound-induced stress in Arabidopsis [80], the identification of flavonoid and isoflavonoid compounds in *Medicago truncatula* [81], the untargeted large-scale analysis of plant metabolome [82], the characterization of metabolites that respond to stress [83, 84], and the identification of flavonoid glycoconjugates in roots form *Medicago truncaluta* [85].

Another technique used for the quantitative analysis of plant metabolites is capillary electrophoresis coupled to MS (CE-MS) [86]. CE-MS was used to characterize the amino acid profile in plant cell cultures [87], to characterize the metabolome of bacterial-infected orange leaves [88], and to identify the metabolite profile in *Illicium anisatum* [89]. CE coupled with MS has also the advantages of high resolution and high reliability [86].

 Fourier transformed infrared spectroscopy (FT-IR), near infrared resonance (NIR) Raman spectroscopy, and, more recently, nuclear magnetic resonance (NMR) spectroscopy are constantly developing rapid, nondestructive and high-throughput techniques for a diverse range of sample types. FT-IR has also been introduced as a metabolic fingerprinting technique within the plant sciences [90, 91]. Raman spectroscopy coupled with microscopy has been recently used with great success, making possible the identification and quantification of phytochemicals and their distribution directly from plant tissues [92–94].

NMR techniques can uniquely identify and simultaneously quantify a wide range of organic compounds in the micromolar range [95, 96]. NMR-based methods can be broadly classified into solution NMR and insoluble or solid-state NMR, according to sample solubility. In onedimensional NMR, protons (^1^H) are usually observed (^1^H-NMR) due to the sensitivity and common occurrence of this magnetic nucleus. More detailed analyses, such as metabolite identification or flux assay, can be obtained with other nuclei, particularly ^13^C and ^15^N that are coupled with ^1^H nuclei in twodimensional or multidimensional NMR analysis [97, 98]. Recently, NMR has been used to identify secondary metabolites involved in host-plant resistance [99] and to characterize the metabolites produced after salt stress exposure in maize plants [100]. Other applications of NMR are in the investigation of food quality and in the standardization and control of different phytomedical preparations [101].

The resulting MS or NMR spectra are preprocessed to suppress noise and align the different peaks. Thereafter, the data are used to identify the different metabolites present in the sample using metabolite databases. In nontarget analyses, the spectra of unknown compounds can be subjected to statistical analyses [102]. In target analyses, the sets of spectral data that are associated with particular compounds are used as metabolic profiles for each compound in further analyses [102].

All the metabolomic technologies mentioned require data processing analysis due to, for example, some inaccuracies such as chromatogram shift and mass drift. Some of the most used methods to group metabolites in samples include multivariate statistical analyses such as principal component analysis (PCA), hierarchical clustering analysis (HCA), and self-organization mapping (SOM) [102]. In addition, the analysis and comprehension of the data obtained by metabolomics is often integrated with data from genomic and proteomic experiments [102].

## 5. Metabolomic Resources

Several metabolomic resources and metabolite databases are available (Table 4). The Human Metabolome Database is an electronic database which contains information on metabolites found in humans (http://www.hmdb.ca/). This database includes chemical, clinical, and biochemical data, linking known metabolites to several genes and proteins. The Golm Metabolome Database (http://gmd.mpimp-golm.mpg.de/) contains information about mass spectra from active metabolites quantified by GS-MS. The Madison Metabolomics Database is a resource for metabolomics research based on NMR and MS (http://mmcd.nmrfam.wisc.edu/). Another integrative database is Metabolights (http://www.ebi.ac.uk/metabolights/). This database is cross-species and contains information about metabolite structures, spectra and biological roles. Metabolomics at Rothamsted (MeT-RO) is an initiative that contains several resources that can be applied to plant and microbial metabolomics (http://www.metabolomics.bbsrc.ac.uk/MeT-RO.htm). The Metlin Metabolite Database contains information on about 55,000 metabolites and nearly 50,000 high resolution MS/MS spectra and tandem MS experiments (http://metlin.scripps.edu/). PRIMe, a platform for RIKEN metabolomics is a database that integrates genomic and metabolomic data. It contains information on metabolites obtained from NMR spectroscopy, GC-MS, LC-MS, and CE-MS (http://prime.psc.riken.jp/).

Several databases for plant species have also been developed, such as Plantmetabolomics (http://plantmetabolomics.vrac.iastate.edu/ver2/). This initiative started as a metabolomic and functional genomic tool for elucidating the functions of Arabidopsis genes. In addition, other databases such as the Metabolome Tomato Database are available and contain information on metabolites identified by LC-MS [79]. A related database is Terpmed (http://www.terpmed.eu/databases.html) which contains information about plant terpenoids, natural products, and other secondary metabolites important for use as therapeutic drugs. The Armec Repository Project was created as a tool to annotate flow injection electrospray MS (FIE-MS) data, but information about HPLC-ESI-MS can also be found in this database. Moreover, at present the Armec database is in expansion, including information about additional species focused on food crops and human metabolome for use in nutrition research (http://www.armec.org/MetaboliteLibrary/).

On the other hand, there are several resources that integrate metabolite data with metabolic pathways such as MetaCyc (http://metacyc.org/) that contains information on about 1800 pathways from more than 2000 organisms. Other related databases are RiceCyc (http://pathway.gramene.org/gramene/ricecyc.shtml), AraCyc (http://www.arabidopsis.org/biocyc/), Solanacea Genomics Network (SolCyc, http://solgenomics.net/tools/solcyc/index.pl), HumanCyc (http://humancyc.org/), KEGG Pathway database (http://www.genome.jp/kegg/pathway.html), and Mapman (http://mapman.gabipd.org/web/guest/mapman).

## 6. Conclusions

Metabolomic studies require knowledge in many areas such as biochemistry, biology, physiology, and bioinformatics. Metabolomics has the potential to make a large impact on different areas of biology, including human health, plant biotechnology, toxicology, and pharmacology, among others. The analysis and comprehension of the data obtained require the use of bioinformatics, as well as the integration with data obtained from genomic and proteomic analyses. 

With the development of more sensitive technologies and also computational tools for statistical analysis and data interpretation, metabolomics has the potential to help us better understand the molecular mechanisms of disease. In addition, the identification and characterization of new biomarkers will allow the diagnosis and prevention of many diseases, as well as the discovery of new drugs. However, in most diseases the analysis of other biomarkers, like protein markers as well as the evaluation of the physiological state for diagnosis and treatment is also required.

In the area of plant biotechnology, metabolomics, together with genomic and proteomic studies, has identified new genes or genes with new functions. In this area, metabolomics has gained importance in the evaluation of transgenic plants, food quality, the increase disease resistance, and herbicide or salinity tolerance. Moreover, the integration of the three *omics* and systems biology is an excellent strategy for the discovery enzymes involved in unknown metabolic pathways. In addition, metabolomics applied to the study of plants becomes extremely important in the efforts that are performed in order to improve human health. As there are several examples where the manipulation of different metabolic pathways led to an increase in the production of a particular metabolite, the modification of plant metabolic pathways could lead to the production of new drugs that could be used for the treatment of many diseases.

## Figures and Tables

**Table 1:** A comparison between the most used methods applied to metabolomic research.

Technique	Use	Overview	References
HPLC-MS	a, b, c	High sensitivity, nonvolatile metabolites, expensive	[12, 13]
GC-MS	a, c	High sensitivity, only volatile compounds, nonexpensive	[14, 15]
NMR	b, d, e	For highly abundant metabolites, nondestructive, very reproducible, low sensitivity	[16]
MS	a, b, c, d, e	High sensitivity, usually coupled with other techniques	[17]
Raman spectroscopy	e	Low resolution, sensitivity, automated, high throughput	[18]

Ref: a: metabolite target analysis; b: metabolite profiling; c: metabolomics; d: metabolite flux analysis; e: metabolic fingerprinting. MS: mass spectrometry; HPLC-MS: high-performance liquid chromatography-MS; GS-MS: gas chromatography-MS; NMR: nuclear magnetic resonance spectroscopy.

**Table 2:** Metabolites used as biomarkers of human diseases.

Disease	Metabolite biomarker	References
Male infertility	Citrate, lactate, and glycerylphosphorylcholine	[23, 24]
Lung cancer	Metabolites that are shown to have a statistically significant difference between healthy individuals and lung cancer patients were hippurate, trigonelline, *β*-hydroxyisovalerate, *α*-hydroxyisobutyrate, N-acetylglutamine, and creatinine	[25]
Alzheimer's disease	Succinic anhydride, pyruvic acid, 2-aminopropanol, n,n-didemethylchlorpromazine, L-alanine n-butyl ester, L-glutamic acid dibutyl ester, L-dopa, taurine, creatine, creatinine, lactate, *β*-alanine, cysteine, fumaric acid, 2-octenedioic acid, and acetoacetic acid	[26, 27]
Respiratory diseases	Asthmatic children: acetate; chronic obstructive pulmonary disease (COPD): leucine, lactate, propionate, acetate, and pyruvate	[28, 29]
Huntington disease	3-Nitropropionic acid	[30]
Multiple sclerosis	Elevated levels: 2-aminobutyrate, 1,3-dimethylurrate, glutamate, and acetate. Reduced levels: oxaloacetate, citrate, alanine, and 3-hydroxybutyrate.	[31]
Impaired glucose tolerance (IGT)	Significantly altered levels: glycine, lysophosphatidylcholine (LPC) (18:2), and acetylcarnitine	[32]
Renal cell carcinoma	Phospholipids, phenylalanine, tryptophan, acylcarnitines, cholesterol metabolites, and arachidonic acid metabolism	[33]
Colorectal cancer	Acteylcarnine, phenylacetylglutamin, leucylproline, and aspartyllysine	[34]
Kidney cancer	Quinolinate, 4-hydroxybenzoate, and gentisate	[34]

**Table 3:** Recent applications of metabolomics in plant biotechnology.

Organism	Application	Technology used	Reference
*Catharanthus roseus *	Improvement of the production of anticancer indole alkaloid by overexpression of ORCA3 and G10H in C. roseus plants	NMR	[68]
*Panicum virgatum* (switchgrass)	Increased amounts of phenolic acids and a monolignol analog associated with more facile cell wall deconstruction	GC-MS	[69]
*Solanum tuberosum* (L)	Increased drought tolerance by expression of trehalose-6-phosphate synthase 1	GC-MS	[70]
*Oryza sativa *	Modulation of salt tolerance by reduction of OsSUT1 (O. sativa sucrose transporter 1) expression	GC-TOF-MS	[71]
*Arabidopsis thaliana *	Distinguish transgenic and nontransgenic plants	NMR	[72]
*Solanum Lycopersicum *	Higher accumulation of flavonoids and thus nutritional value in tomato plants carrying a mutation in HP1/LeDDB1 gene	LC-ESI-MS/MS	[73]

**Table 4:** Metabolomic resources.

Name	URL	Information/Species
Human metabolome database	http://www.hmdb.ca/	Chemical and biological data of human metabolites
Golm metabolome database	http://gmd.mpimp-golm.mpg.de/	GS-MS
Madison metabolomics database	http://mmcd.nmrfam.wisc.edu/	NMR and MS
Metabolights	http://www.ebi.ac.uk/metabolights/	Metabolite structures, spectra, function/cross-species
Metabolomics at Rothamsted (MeT-RO)	http://www.metabolomics.bbsrc.ac.uk/MeT-RO.htm	Plant and microbial metabolites
Metlin metabolite database	http://metlin.scripps.edu/	High resolution MS/MS spectra and tandem MS experiments
PRIMe	http://prime.psc.riken.jp/	Genomic and metabolomics data/NMR spectroscopy, GC-MS, LC-MS and CE-MS
Plantmetabolomics	http://plantmetabolomics.vrac.iastate.edu/ver2/	Arabidopsis and other plant species
Metabolome tomato database	[79]	Metabolites identified by LC-MS
Terpmed	http://www.terpmed.eu/databases.html	Plant terpenoids, natural products, secondary metabolites/Therapeutic drugs
Armec repository project	http://www.armec.org/MetaboliteLibrary/	FIE-MS/HPLC-ESI-MS data/human and plant metabolomes for nutrition
MetaCyc	http://metacyc.org/	Integration of metabolite data with metabolic pathways/2000 organisms
RiceCyc	http://pathway.gramene.org/gramene/ricecyc.shtml	Metabolic pathways, enzymes, metabolites
AraCyc	http://www.arabidopsis.org/biocyc/	Metabolic pathways, compounds/Arabidopsis
Solanacea Genomics network (SolCyc)	http://solgenomics.net/tools/solcyc/index.pl	Pathway genome databases/solanacea species
HumanCyc	http://humancyc.org/	Metabolic pathways, genome/human
KEGG Pathway database	http://www.genome.jp/kegg/pathway.html	Pathways, metabolism, genetic information Cellular processes, human diseases
Mapman	http://mapman.gabipd.org/web/guest/mapman	Datasets (e.g., Gene expression data, metabolic pathways)
